# Repeat polymorphisms in *ESR2* and *AR* and colorectal cancer risk and prognosis: results from a German population-based case-control study

**DOI:** 10.1186/1471-2407-14-817

**Published:** 2014-11-07

**Authors:** Anja Rudolph, Hong Shi, Asta Försti, Michael Hoffmeister, Juan Sainz, Lina Jansen, Kari Hemminki, Hermann Brenner, Jenny Chang-Claude

**Affiliations:** Division of Cancer Epidemiology, German Cancer Research Center (DKFZ), Im Neuenheimer Feld 581, 69120 Heidelberg, Germany; Division of Molecular Genetic Epidemiology, German Cancer Research Center (DKFZ), Im Neuenheimer Feld 580, 69120 Heidelberg, Germany; Department of Oncology, Tangdu Hospital of Fourth Military Medical University, Changle West Rd, Xi’an, 710032 People’s Republic of China; Center for Primary Health Care Research, Clinical Research Center, Jan Waldenströms gata 35, SUS, 205 02 Malmö, Sweden; Division of Clinical Epidemiology and Aging Research, German Cancer Research Center (DKFZ), Im Neuenheimer Feld 581, 69120 Heidelberg, Germany; Pfizer - Universidad de Granada - Junta de Andalucía Centre for Genomics and Oncological Research (GENYO), Av de la Ilustración 114, 18007 Granada, Spain; German Cancer Consortium (DKTK), Im Neuenheimer Feld 280, 69120 Heidelberg, Germany

**Keywords:** Colorectal cancer, Estrogen receptor beta, Androgen receptor, Genetic polymorphism, Short tandem repeat

## Abstract

**Background:**

Evidence has accumulated which suggests that sex steroids influence colorectal cancer development and progression. We therefore assessed the association of repeat polymorphisms in the estrogen receptor β gene (*ESR2*) and the androgen receptor gene (*AR*) with colorectal cancer risk and prognosis.

**Methods:**

The *ESR2* CA and *AR* CAG repeat polymorphisms were genotyped in 1798 cases (746 female, 1052 male) and 1810 controls (732 female, 1078 male), matched for sex, age and county of residence. Colorectal cancer risk associations overall and specific for gender were evaluated using multivariate logistic regression models adjusted for sex, county of residence and age. Associations with overall and disease-specific survival were evaluated using Cox proportional hazard models adjusted for established prognostic factors (diagnosis of other cancer after colorectal cancer diagnosis, detection by screening, treatment with adjuvant chemotherapy, tumour extent, nodal status, distant metastasis, body mass index, age at diagnosis and year of diagnosis) and stratified for grade of differentiation. Heterogeneity in gender specific associations was assessed by comparing models with and without a multiplicative interaction term by means of a likelihood ratio test.

**Results:**

The average number of *ESR2* CA repeats was associated with a small 5% increase in colorectal cancer risk (OR = 1.05, 95% CI 1.01-1.10) without significant heterogeneity according to gender or tumoural ESR2 expression. We found no indication for an association between the *AR* CAG repeat polymorphisms and risk of colorectal cancer. The *ESR2* CA and *AR* CAG repeat polymorphisms were not associated with overall survival or disease specific survival after colorectal cancer diagnosis.

**Conclusions:**

Higher numbers of *ESR2* CA repeats are potentially associated with a small increase in colorectal cancer risk. Our study does not support an association between colorectal cancer prognosis and the investigated repeat polymorphisms.

**Electronic supplementary material:**

The online version of this article (doi:10.1186/1471-2407-14-817) contains supplementary material, which is available to authorized users.

## Background

Colorectal cancer is increasingly being recognized as a hormone related disease due to accumulating evidence that sex steroids influence colorectal carcinogenesis and prognosis [1]. Incidence rates of colorectal cancer are lower in women than in men and the use of menopausal hormone therapy has consistently found to be associated with a reduced colorectal cancer risk [2, 3]. The effects of sex hormones are potentially exerted through the respective nuclear receptors. In normal colorectal tissue, the estrogen receptor β (ESR2) is the predominantly expressed estrogen receptor, and estrogen receptor α, which plays a major role in breast cancer development and therapy [4], is expressed at very low levels [1]. Another nuclear hormone receptor expressed in colorectal tissue is the androgen receptor (AR) [5, 6]. Both receptors translate hormonal stimuli into transcriptional changes, leading to specific modifications in gene expression [7, 8].

A CA repeat exists in intron 5 of *ESR2*, and it was found to be associated with serum androgen, sex hormone-binding globulin (SHBG) and estradiol levels [9, 10]. Similarly, associations between the CAG repeat in exon 1 of the X-linked *AR* with serum testosterone and estradiol levels in men were observed [11–13]. The number of *AR* CAG repeats was shown to have functional implications on the resulting protein with higher numbers leading to decreased transcriptional activity [14, 15]. Both polymorphisms were found to be associated with colon cancer risk in a previous study [16]. Women harbouring two long alleles (≥25 CA repeats) of the *ESR2* CA repeat and men having two alleles with ≥23 CAG repeats in *AR* were at increased risk for colon cancer compared to individuals with shorter alleles of the respective polymorphism. Recent studies on prognosis observed that men with metastatic colorectal cancer harbouring two long alleles of the *ESR2* CA repeat had poorer overall and progression-free survival than men with short alleles [17] and women with metastatic colon cancer harbouring two long alleles had a significantly reduced risk of dying compared to women with at least one short allele [18]. Furthermore, loss of ESR2 expression in colorectal tumours was associated with an increased risk of mortality [19]. Therefore, genetic variation in *ESR2* and *AR* may affect the action of sex steroids on colonic epithelium and consequently influence the colorectal cancer susceptibility and prognosis. Whether the *AR* CAG repeat is associated with colorectal cancer prognosis has not been investigated so far.

With the present study, we aimed to investigate the association between the *AR* CAG and *ESR2* CA repeat polymorphism and colorectal cancer risk and prognosis, also stratified by the tumoural expression of ESR2. In order to compare our results with those previously published, the analyses were also conducted separately in men and women.

## Methods

### Study sample, data collection and follow-up

The DACHS study is an ongoing population-based case-control study conducted in southwest Germany, which has previously been described in detail [20, 21]. Briefly, cases were recruited from patients who received in-patient treatment in a hospital of the study region due to a first diagnosis of colorectal cancer. To be eligible, participants had to be at least 30 years old and capable to complete the interview. Controls were randomly selected from lists of population registries and matched according to gender, 5-year age groups and county of residence. Individuals with a history of colorectal cancer were excluded from the study. The present study comprised 746 female and 1052 male incident colorectal cancer patients as well as 732 female and 1078 male controls recruited between January 01, 2003 and December 31, 2007. Ancestry of the participants was homogenous with about 1% being of non-European descent. Patients diagnosed with any other cancer except squamous and basal cell skin cancer before their first diagnosis of colorectal cancer (N =160), patients who died within 30 days after diagnosis and whose death may be related to surgery (N =7), and patients without follow-up information (N =7) were excluded from the survival analyses, which comprised 665 female and 959 male cases. Written informed consent was obtained from all study participants and the study was approved by the ethics committees of the University of Heidelberg and the State Medical Boards of Baden-Wuerttemberg and Rhineland-Palatinate, Germany.

Patients and controls were interviewed in person by trained interviewers using standardized questionnaires. In the interview, information on sociodemographic factors, previous health examinations, medication such as the use of menopausal hormone therapy and non-steroidal anti-inflammatory drugs (NSAIDs), family history of colorectal cancer, and life-style related factors was collected. Additionally, pathology reports and discharge letters were collected. Self-reported use of menopausal hormone therapy was validated for women entering the study before December 31, 2006 [22]. The study participants were asked to provide either a blood sample or a mouthwash sample.

On average three years after diagnosis, a questionnaire was sent to the treating physicians of the patients to collect information on therapy, and newly diagnosed concomitant diseases. A second follow-up questionnaire was mailed to the patients about five years after diagnosis. Vital status and date of death were obtained from the population registries and the cause of death was verified by death certificates obtained from the health authorities in the Rhein-Neckar-Odenwald region. New diagnoses and cancer recurrences were verified through medical records of the attending physicians. In total 665 female and 959 male cases were included in the survival analysis.

### Genotyping

Genomic DNA was extracted from blood (98%) or mouthwash samples (2%) using Flexigene Kit 250 (Qiagen, Valencia, CA, USA) and Qiagen Mini Kit (Qiagen, Valencia, CA, USA), respectively. Genomic regions containing the *AR* and *ESR2* microsatellite markers were amplified by polymerase chain reaction (PCR). We used previously reported primers [9]. The PCR reaction mixture consisted of 4 ng genomic DNA in a 5 μl reaction volume containing 1.5 mM magnesium chloride, 1x reaction buffer, 0.20 μM deoxynucleoside triphosphates mixture, 0.18U Platinum-Taq DNA polymerase (Invitrogen, Darmstadt, Germany) and 0.5 μM of each primer. About 2 μl of the 1/10 diluted PCR product was added to 0.5 μl size marker and denaturized for 3 minutes at 95°C. The detection was done using the ABI 3130XL Genetic Analyzer (Applied Biosystems, Carlsbad, CA, USA) and the fluorescently labelled DNA fragments were analysed by size using the GeneMapper 4.0 software (Applied Biosystems, Carlsbad, CA, USA). A random sample of 6.6% was genotyped twice for quality control.

### Definition of variables

The repeat numbers in *AR* and *ESR2* were averaged for each individual, assuming that each increase in number is related to a constant proportional change in relative risk. For better illustration of the associations with differing levels of repeat number, we categorized the continuous variable of average repeat number into quartiles, according to the distribution in controls. The genotypes were also dichotomized in order to report results comparable to previous studies, based on the median repeat number in controls (<22 repeats/≥22 for *AR* CAG repeats and <24 repeats/≥24 for *ESR2* CA repeats).

To evaluate the ESR2-status of colorectal tumours, ESR2 expression was measured immunohistochemically in tissue microarrays [19]. For this study, we classified samples with less than 10% of the cell nuclei showing strong positive staining or with less than 50% of the nuclei showing weak positive staining as ESR2 negative. ESR2 positivity was defined as weak staining of more than 50% of the cell nuclei or strong positive staining in at least 10% of the cell nuclei.

For survival analysis, follow-up time was calculated as the time between the date of diagnosis and the date of event or censoring. Events of interest were death from any cause (overall survival) and death due to colorectal cancer (disease-specific survival).

### Statistical analysis

All statistical analyses were performed using SAS 9.2 (SAS Institute, Cary, NC, USA). Statistical significance was determined according to the conventional significance-level of α =5%.

Genotype frequencies were assessed in cases and controls separately and tested for deviation from Hardy-Weinberg equilibrium (HWE) in controls using a one degree of freedom Chi-square test. Unconditional logistic regression was used to calculate odds ratios (ORs) as well as confidence intervals (CIs) for colorectal cancer risk associated with genotypes. To test whether gender specific associations are statistically different, we built a multiplicative interaction term between the respective genotype variable and gender and performed a log likelihood ratio test. The models were adjusted for age and county of residence. The inclusion of additional colorectal cancer risk factors did not change the OR estimates substantially (changes <10% in all cases). The following factors were assessed: having a first degree relative diagnosed with colorectal cancer, ever regular use of NSAIDs (2+ times/week, ≥1 year), pack-years of smoking (in categories of 10 pack-years), average lifetime alcohol consumption (g/day in quartiles), average physical activity in the 12 months before diagnosis (in metabolic equivalent of task (MET) hours/week quartiles), ever colorectal endoscopy, ever diagnosis of diabetes, consumption of red meat in last 12 months (low, moderate, high) and body mass index (BMI) ≥5 years before diagnosis/date of interview (in five categories, <23 kg/m^2^, ≥23 to <25 kg/m^2^, ≥25 to <27 kg/m^2^, ≥27 to <30 kg/m^2^, ≥30 kg/m^2^). In secondary analysis, we evaluated risk associations according to ESR2-status using multinomial logistic regression. Heterogeneity between the risk estimates was assessed using unadjusted logistic regression models in case-case analyses.

Median follow-up time of cases after diagnosis was computed using the reverse Kaplan-Meier method [23]. Regression analyses based on the Cox proportional hazards models were applied to evaluate associations of the polymorphisms with overall and disease-specific survival. The models were determined using backward selection, retaining variables with a *p*-value of ≤0.2. Validity of the proportional hazards assumption was assessed by including a time-dependent component for each explanatory variable. The models were adjusted for tumour extent (T1, T2, T3, T4), nodal status (N0, N1, N2), distant metastasis (M0, M1), screening detection of colorectal cancer (yes/no), treatment with adjuvant chemotherapy (yes/no), BMI at diagnosis (kg/m^2^, continuous), diagnosis of diabetes after colorectal cancer diagnosis (yes/no), diagnosis of other cancer after colorectal cancer diagnosis (yes/no), age at diagnosis and year of diagnosis. The models were additionally stratified for grade of differentiation (well/moderate, poor/undifferentiated) as this variable showed a time-dependent effect on overall survival. We accounted for left truncation of the follow-up period. The association of the *ESR2* and *AR* repeat polymorphisms with survival according to tumoural ESR2-status was assessed using subgroup analysis. Heterogeneity of ESR2-specific hazard ratios was evaluated using an interaction term between ESR2-status and genotype.

## Results

**Figure 1:**
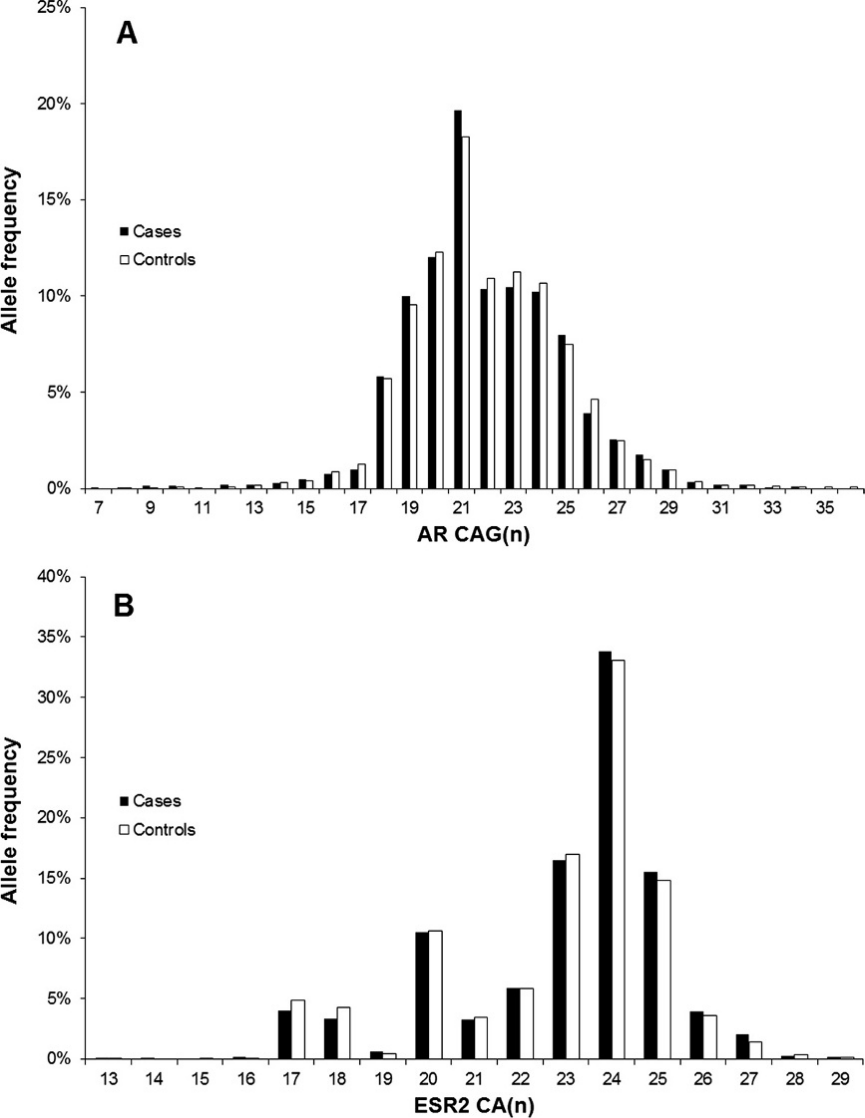
**Frequencies of the average number of (A) CAG repeats in** ***AR*** **and (B) CA repeats in** ***ESR2*** **.**

The distribution of relevant epidemiologic characteristics for women and men are shown in Additional file 1: Table S1. The median follow-up time was 48.4 months in men and 49.9 months in women. For the *AR* CAG repeat, the genotype was successfully determined in 89.4% of cases and 87.7% of controls. The genotyping error rate calculated from the duplicated samples was 4.1%. Because the AR gene is X-linked, a heterozygous genotype among men indicates a genotyping error. The respective samples (20 cases, 16 controls) were excluded from further analyses. For the *ESR2* CA repeat, genotyping was successful in 87.9% of cases and 89.4% of controls. The genotyping error rate was 0.8%. The distribution of genotypes among controls did not significantly deviate from HWE for any of the investigated variants, although this could not be assessed for the *AR* CAG repeat among male controls (HWE *p*-value was 0.14 for the *AR* CAG repeat and 0.98 for the *ESR2* CA repeat). The allele frequencies of the *AR* CAG and the *ESR2* CA repeats are shown in Figure 1.

The estimated ORs and 95% CIs for colorectal cancer risk associated with the average number of the *AR* CAG repeats and *ESR2* CA repeats are displayed in Table 1. The number of CAG repeats in *AR* was not associated with colorectal cancer risk. A significant positive association with colorectal cancer risk was found with average number of the CA repeats in *ESR2* (OR per unit increase in average repeat number =1.05, 95% CI 1.01 - 1.10, *p* =0.02). The association was significant in men (OR =1.07, 95% CI 1.02 - 1.13, *p* =0.01) and not apparent in women (OR =1.01, 95% CI 0.95 - 1.08, *p* =0.69), although the *p*-value for heterogeneity by gender did not indicate a significantly heterogeneous association between men and women (*p* heterogeneity =0.24). Associations of the repeat polymorphisms with colorectal cancer risk did not differ for ESR2 positive and ESR2 negative cancer (Additional file 1: Table S2).

**Table 1:** **Number of** ***AR*** **CAG repeats and** ***ESR2*** **CA repeats and associated colorectal cancer risk in the female and male study population**

	Overall			Women			Men			
Genotype	Cases N	Controls N	OR (95% CI) ^b^	Cases N	Controls N	OR (95% CI) ^c^	Cases N	Controls N	OR (95%CI) ^c^	***p***heterogeneity
***AR*** **CAG(** **n)**										
<20 average repeats	263	254	1.00 (Ref.)	88	94	1.00 (Ref.)	175	160	1.00 (Ref.)	
≥20 to <22 average repeats	531	506	1.01 (0.82 - 1.25)	229	228	1.06 (0.75 - 1.51)	302	278	1.00 (0.76 - 1.31)	
≥22 to <23.5 average repeats	370	400	0.89 (0.71 - 1.11)	182	189	1.03 (0.72 - 1.48)	188	211	0.82 (0.61 - 1.10)	
≥23.5 average repeats	424	412	0.99 (0.79 - 1.23)	169	140	1.24 (0.85 - 1.80)	255	272	0.86 (0.65 - 1.13)	0.19^d^
average repeats^a^	1588	1572	0.99 (0.96 - 1.01)	668	651	1.01 (0.96 - 1.06)	920	921	0.98 (0.95 - 1.01)	0.23^e^
			*p* trend =0.34			*p* trend =0.68			*p* trend =0.15	
<22/<22 repeats	645	610	1.00 (Ref.)	168	172	1.00 (Ref.)	477	438	1.00 (Ref.)	
<22/≥22 repeats	324	324	0.91 (0.72 - 1.15)	324	324	1.03 (0.79 - 1.35)	n/a	n/a	(no heterozygous)	
≥22/≥22 repeats	619	638	0.91 (0.78 - 1.07)	176	155	1.14 (0.84 - 1.55)	443	483	0.84 (0.70 - 1.01)	0.09^e^
			*p* trend =0.24			*p* trend =0.40			*p* trend =0.07	
***ESR2*** **CA(** **n)**										
<22 average repeats	348	409	1.00 (Ref.)	149	153	1.00 (Ref.)	199	256	1.00 (Ref.)	
≥22 to <23.5average repeats	389	381	1.20 (0.98 - 1.47)	154	154	1.07 (0.77 - 1.49)	235	227	1.25 (0.96 - 1.64)	
≥23.5 to <24 average repeats	226	227	1.17 (0.93 - 1.48)	98	90	1.14 (0.77 - 1.68)	128	137	1.20 (0.87 - 1.65)	
≥24 average repeats	618	602	1.20 (1.00 - 1.45)	261	255	1.06 (0.78 - 1.46)	357	347	1.31 (1.02 - 1.70)	0.51^d^
average repeats^a^	1581	1619	1.05 (1.01 - 1.10)	662	652	1.01 (0.95 - 1.08)	919	967	1.07 (1.02 - 1.13)	0.24^e^
			*p* trend =0.02			*p* trend =0.69			*p* trend =0.01	
<24/<24 repeats	323	347	1.00 (Ref.)	119	125	1.00 (Ref.)	204	222	1.00 (Ref.)	
<24/≥24 repeats	755	814	0.99 (0.83 - 1.19)	335	334	1.03 (0.77 - 1.39)	420	480	0.95 (0.75 - 1.20)	
≥24/≥24 repeats	503	458	1.17 (0.96 - 1.43)	208	193	1.10 (0.80 - 1.52)	295	265	1.21 (0.94 - 1.55)	0.71^e^
			*p* trend =0.08			*p* trend =0.54			*p* trend =0.11	

^a^As continuous variable, ^b^Models adjusted for sex, county of residence and age, ^c^Models adjusted for county of residence and age, ^d^ *P* value for heterogeneity by gender with genotype in categories (3 df), ^e^ *P* value for heterogeneity by gender with genotype as continuous variable (1 df), OR: odds ratio, CI: confidence interval.

The *AR* CAG and *ESR2* CA repeat polymorphisms were not associated with overall or disease-specific survival for all stages of colorectal cancer in multivariate analyses and the associations were not significantly different in men and women. The respective hazard ratios (HRs) and CIs are presented in Table 2. No significant associations between prognosis and the investigated polymorphisms were observed when assessing hazard ratios according to ESR2-status of the tumour (Additional file 1: Table S3).

**Table 2:** **Associations between number of** ***AR*** **CAG repeats and** ***ESR2*** **CA repeats and overall as well as disease**-**specific survival**

	Overall		Women		Men		***p***heterogeneity ^c^	
Genotype	OS HR (95%CI) ^b^	DSS HR (95%CI) ^b^	OS HR (95% CI) ^b^	DSS HR (95%CI) ^b^	OS HR (95%CI) ^b^	DSS HR (95% CI) ^b^	OS	DSS
***AR*** **CAG(** **n)**								
<20 average repeats	1.00 (Ref.)	1.00 (Ref.)	1.00 (Ref.)	1.00 (Ref.)	1.00 (Ref.)	1.00 (Ref.)		
≥20 to <22 average repeats	1.09 (0.78 - 1.53)	1.14 (0.77 - 1.69)	0.90 (0.50 - 1.62)	1.21 (0.59 - 2.47)	1.28 (0.84 - 1.97)	1.20 (0.73 - 2.00)		
≥22 to <23.5 average repeats	1.11 (0.76 - 1.61)	1.26 (0.82 - 1.95)	0.90 (0.49 - 1.64)	1.17 (0.56 - 2.46)	1.12 (0.67 - 1.88)	1.34 (0.74 - 2.42)		
≥23.5 average repeats	1.03 (0.72 - 1.46)	1.04 (0.69 - 1.58)	0.75 (0.41 - 1.37)	0.93 (0.44 - 1.96)	1.22 (0.78 - 1.91)	1.24 (0.72 - 2.12)	0.83^c^	0.99^c^
average repeats^a^	1.00 (0.96 - 1.05)	1.01 (0.95 - 1.06)	0.96 (0.88 - 1.04)	0.98 (0.89 - 1.08)	1.02 (0.96 - 1.07)	1.03 (0.96 - 1.10)	0.45^d^	0.93^d^
	*p* trend =0.97	*p* trend =0.84	*p* trend =0.31	*p* trend =0.67	*p* trend =0.56	*p* trend =0.46		
<22/<22 repeats	1.00 (Ref.)	1.00 (Ref.)	1.00 (Ref.)	1.00 (Ref.)	1.00 (Ref.)	1.00 (Ref.)		
<22/≥22 repeats	0.95 (0.70 - 1.28)	0.95 (0.66 - 1.36)	0.96 (0.62 - 1.49)	0.97 (0.59 - 1.60)	(no heterozygous)	(no heterozygous)		
≥22/≥22 repeats	1.03 (0.80 - 1.33)	1.12 (0.84 - 1.51)	0.94 (0.58 - 1.53)	1.09 (0.63 - 1.89)	1.01 (0.74 - 1.37)	1.14 (0.78 - 1.65)	0.91^d^	0.60^d^
	*p* trend =0.80	*p* trend =0.45	*p* trend =0.82	*p* trend =0.73	*p* trend =0.95	*p* trend =0.50		
***ESR2*** **CA(** **n)**								
<22 average repeats	1.00 (Ref.)	1.00 (Ref.)	1.00 (Ref.)	1.00 (Ref.)	1.00 (Ref.)	1.00 (Ref.)		
≥22 to <23.5average repeats	0.82 (0.59 - 1.15)	0.92 (0.61 - 1.39)	0.66 (0.38 - 1.15)	0.79 (0.42 - 1.47)	0.76 (0.51 - 1.14)	1.11 (0.63 - 1.97)		
≥23.5 to <24 average repeats	0.93 (0.64 - 1.35)	1.12 (0.73 - 1.73)	1.15 (0.67 - 1.98)	1.16 (0.61 - 2.20)	0.91 (0.54 - 1.53)	1.30 (0.69 - 2.44)		
≥24 average repeats	0.76 (0.56 - 1.02)	0.91 (0.64 - 1.29)	0.73 (0.47 - 1.15)	0.80 (0.50 - 1.38)	0.83 (0.50 - 1.38)	1.00 (0.60 - 1.65)	0.56^c^	0.96^c^
average repeats^a^	0.95 (0.89 - 1.02)	0.99 (0.91 - 1.07)	0.95 (0.86 - 1.06)	0.96 (0.86 - 1.08)	0.95 (0.86 - 1.03)	1.00 (0.89 - 1.12)	0.85^d^	0.81^d^
	*p* trend =0.14	*p* trend =0.72	*p* trend =0.35	*p* trend =0.54	*p* trend =0.22	*p* trend =0.99		
<24/<24 repeats	1.00 (Ref.)	1.00 (Ref.)	1.00 (Ref.)	1.00 (Ref.)	1.00 (Ref.)	1.00 (Ref.)		
<24/≥24 repeats	0.79 (0.59 - 1.07)	0.88 (0.62 - 1.26)	0.74 (0.46 - 1.20)	0.89 (0.51 - 1.55)	0.88 (0.60 - 1.29)	0.99 (0.62- 1.58)		
≥24/≥24 repeats	0.73 (0.53 - 1.00)	0.82 (0.57 - 1.19)	0.67 (0.40 - 1.12)	0.78 (0.43 - 1.40)	0.76 (0.51 - 1.15)	0.89 (0.54 - 1.46)	0.81^d^	0.84^d^
	*p* trend =0.06	*p* trend =0.31	*p* trend =0.15	*p* trend =0.39	*p* trend =0.19	*p* trend =0.62		

^a^As continuous variable, ^b^Stratified for grade of differentiation (well/moderate, poor/undifferentiated) and adjusted for diagnosis of other cancer after colorectal cancer diagnosis (yes/no), colorectal cancer detected by screening (yes/no), treatment with adjuvant chemotherapy (yes/no), tumour extent (T1, T2, T3, T4), nodal status (N0, N1, N2), distant metastasis (M0, M1), BMI (kg/m2, continuous), age at diagnosis and year of diagnosis, ^c^P-value for heterogeneity by gender with genotype in categories (3df), ^d^P-value for heterogeneity by gender with genotype as continuous variable (1df), OS: overall survival, DSS: disease-specific survival, HR: hazard ratio, CI: confidence interval.

## Discussion

In the present population-based case-control study, the average number of CA repeats in *ESR2* was positively associated with colorectal cancer risk. We did not observe significant associations between the number of CAG repeats in *AR* and colorectal cancer risk. Regarding colorectal cancer prognosis, the CA repeat polymorphism in *ESR2* and the CAG repeat polymorphism in *AR* were not associated with overall or disease-specific survival.

The *ESR2* repeat polymorphism (having two alleles of ≥25 CA repeats versus 24 CA repeats) was previously reported to be associated with increased risk of colon cancer among women, but not among men (OR women = 2.1 95% CI 1.2 - 3.6, OR men =1.0, 95% CI 0.6 - 1.6, *p* heterogeneity =0.03) [16]. We found a similar association of increasing repeat number with increased risk of colorectal cancer, although significantly so in the overall study population and without significantly different associations according to gender. In contrast to the associations observed here and by Slattery et al., a Japanese study reported a more than six-fold increased risk of colorectal cancer for women harbouring two *ESR2* short alleles (<22 repeats) compared to women harbouring two long alleles (≥22 repeats) [24]. Yet in another independent study, having two *ESR2* alleles with ≥22 CA repeats compared to having two shorter *ESR2* alleles with <22 CA repeats was associated with an increased risk of colon cancer among Japanese women [25]. The discrepancy of the obtained results may be due to chance in light of the relatively small number of cases investigated in both Japanese studies (61 female colorectal cases and 151 female colon cases, respectively) and differences in allele frequencies by ethnicity.

Two studies reported gender-specific associations of the *ESR2* CA repeat polymorphism with overall survival among patients with metastatic colorectal and colon cancer. In the study by Gordon et al., men with two long alleles (≥22 repeats) had poorer overall and progression-free survival than men with at least one short allele (<22 repeats) [17]. Press et al. reported the same association for men, but found further evidence for an opposite association among women [18]. In metastatic colorectal cancer patients of the present study, there was no association between the *ESR2* CA repeat polymorphism and overall survival overall or by gender (data not shown). In addition, an association between tagging SNPs in the promoter region of *ESR2* and an improved overall survival after a diagnosis of colorectal cancer has been reported by Passarelli et al. based on five prospective case-cohorts [26]. Compared to our study, the patient sample analysed by Passarelli et al. had similar 5-year overall survival and distribution of tumour characteristics, but longer median follow-up after diagnosis (5.0 to 9.1 years). Taking into account the reported associations and given that the expression of ESR2 in tumour tissue of colorectal cancer patients has been associated with overall survival [19, 27], it cannot be ruled out that genetic variation influencing ESR2 expression plays a role in colorectal cancer prognosis.

Estrogens are known to regulate the proliferation and differentiation of breast, endometrial and various other tissues [28]. Experimental studies indicate that this is also true for the colonic epithelium [1, 29–31]. Estrogen signalling in the colon is most likely mediated by ESR2, which is highly expressed in both colon epithelial cell lines and human colon epithelium tissue samples [1, 32]. A lack of ESR2 expression in human colon adenocarcinoma has been reported, suggesting that ESR2 might be a tumour suppressor [19, 33–35]. However, our results do not support a differential association of the *ESR2* CA repeat with colorectal cancer risk or prognosis according to ESR2 status. A functional study by Ugai et al. indicated that the number of CA repeats in ESR2 has no effect on *ESR2* transcription [36]. The CA repeat polymorphism in *ESR2* may therefore predominantly affect other processes such as splicing and translation of *ESR2* RNA or ESR2 signalling.

Regarding the relationship between colorectal cancer risk and the *AR* CAG repeat polymorphism, Slattery et al. [16] reported an increased risk for colon cancer for men having two alleles with 23 CAG repeats or more. Their finding is not supported by the present investigation in which the number of CAG repeats in *AR* was not associated with colorectal cancer risk in men or in women. This study investigated for the first time the association between the *AR* CAG repeat polymorphism and colorectal cancer prognosis and did not find a significant association with overall or disease-specific survival. However, the genotyping error rate calculated from the duplicated samples was relatively high for the *AR* CAG repeat polymorphism (4.1%). The misclassification due to genotyping error may have affected study power when investigating associations with the *AR* CAG repeat polymorphism [37].

## Conclusions

In summary, alleles with higher numbers of *ESR2* CA repeats are potentially associated with a small increase in colorectal cancer risk. Further large epidemiological studies as well as functional studies are needed to elucidate the role of *ESR2* and *AR* polymorphisms in colorectal cancer development and prognosis.

## Electronic supplementary material

Additional file 1: Table S1: Distribution of selected risk and preventive factors for colorectal cancer in the female and male study population. **Table S2.** Number of CAG repeats in *AR* and CA repeats in *ESR2* and associated risk for ESR2 positive and ESR2 negative colorectal cancer in the female and male study population. **Table S3.** Associations between number of *AR* CAG repeats and *ESR2* CA repeats and overall as well as disease specific survival according to tumoral ESR2 expression. (DOC 340 KB)
