# Supplementary material for: Repeat polymorphisms in ESR2 and AR and colorectal cancer risk and prognosis: results from a German population-based case-control study
Source: BMC Cancer. 2014 Nov 7;14:817. doi: 10.1186/1471-2407-14-817 (PMC4232639; doi:10.1186/1471-2407-14-817)
Supplement: Supplementary file 1 — Additional file 1: Table S1: Distribution of selected risk and preventive factors for colorectal cancer in the female and male study population. Table S2. Number of CAG repeats in AR and CA repeats in ESR2 and associated risk for ESR2 positive and ESR2 negative colorectal cancer in the female and male study population. Table S3. Associations between number of AR CAG repeats and ESR2 CA repeats and overall as well as disease specific survival according to tumoral ESR2 expression. (DOC 340 KB) [file 12885_2014_4986_MOESM1_ESM.doc]

**Additional file 1**

**Table S1:** Distribution of selected risk and preventive factors for colorectal cancer in the female and male study population.

| **Women** | | | | | | |  | | **Men** | | | | | | | | | | | |
| --- | --- | --- | --- | --- | --- | --- | --- | --- | --- | --- | --- | --- | --- | --- | --- | --- | --- | --- | --- | --- |
| **Characteristic** | **Cases N (%)** | **Controls N (%)** | ***p*** | **Alive^a^ N (%)** | **Dead^a^ N (%)** | ***p*** |  | | **Characteristic** | | **Cases N (%)** | | **Controls N (%)** | | ***p*** | | **Alive^a^ N (%)** | | **Dead^a^ N (%)** | ***p*** |
|  |  |  |  |  |  |  |  | |  | |  | |  | |  | |  | |  |  |
| ***Total*** | 746 (100.0) | 732 (100.0) |  | 482 (72.5) | 183 (27.5) |  |  | |  | | 1052 (100.0) | | 1078 (100.0) | |  | | 732 (76.3) | | 227 (23.7) |  |
|  | | | | | | | | | | | | | | | | | | | | |
| ***Age (years)*** | | | | | | | | | | | | | | | | | | | | |
| 30 to <40 | 7 (0.9) | 2 (0.3) | 0.13 | 4 (0.8) | 2 (1.1) | <0.0001 |  | | 30 to <40 | | 10 (1.0) | | 3 (0.3) | | <0.0001 | | 8 (1.1) | | 2 (0.9) | <0.0001 |
| ≥40 to <50 | 30 (4.0) | 20 (2.7) |  | 20 (4.1) | 9 (4.9) |  |  | | 40 to <50 | | 28 (2.7) | | 40 (3.7) | |  | | 20 (2.7) | | 6 (2.6) |  |
| ≥50 to <60 | 103 (13.8) | 93 (12.7) |  | 75 (15.6) | 19 (10.4) |  |  | | 50 to <60 | | 167 (15.9) | | 167 (15.5) | |  | | 139 (19.0) | | 21 (9.3) |  |
| ≥60 to <70 | 229 (30.7) | 260 (35.5) |  | 156 (32.4) | 45 (24.6) |  |  | | 60 to <70 | | 396 (37.6) | | 315 (29.2) | |  | | 280 (38.3) | | 87 (38.3) |  |
| ≥70 to <80 | 232 (31.1) | 231 (31.6) |  | 158 (32.8) | 50 (27.3) |  |  | | 70 to <80 | | 337 (32.0) | | 384 (35.6) | |  | | 230 (31.4) | | 68 (30.0) |  |
| ≥80 | 145 (19.4) | 126 (17.2) |  | 69 (14.3) | 58 (31.7) |  |  | | 80+ | | 114 (10.8) | | 169 (15.7) | |  | | 55 (7.5) | | 43 (18.9) |  |
|  | | | | | | | | | | | | | | | | | | | | |
| ***Body mass index (kg/m^2^) ≥5 years before diagnosis/date of interview*** | | | | | | | | | | | | | | | | | | | | |
| <23 | 179 (24.0) | 201 (27.5) | 0.001 | 119 (24.7) | 41 (22.4) | 0.52 |  | | <23 | | 79 (7.5) | | 110 (10.2) | | 0.008 | | 57 (7.8) | | 18 (7.9) | 0.26 |
| ≥23 to <25 | 142 (19.0) | 162 (22.1) |  | 96 (19.9) | 40 (21.9) |  |  | | 23 to <25 | | 185 (17.6) | | 224 (20.8) | |  | | 131 (17.9) | | 35 (15.4) |  |
| ≥25 to <27 | 115 (15.4) | 143 (19.5) |  | 78 (16.2) | 20 (10.9) |  |  | | 25 to <27 | | 265 (25.2) | | 286 (26.5) | |  | | 194 (26.5) | | 47 (20.7) |  |
| ≥27 to <30 | 133 (17.8) | 120 (16.4) |  | 84 (17.4) | 31 (16.9) |  |  | | 27 to <30 | | 317 (30.1) | | 288 (26.7) | |  | | 215 (29.4) | | 76 (33.5) |  |
| ≥30 | 157 (21.0) | 102 (13.9) |  | 97 (20.1) | 41 (22.4) |  |  | | >30 | | 198 (18.8) | | 164 (15.2) | |  | | 130 (17.8) | | 49 (21.6) |  |
| unknown | 20 (2.7) | 4 (0.5) |  | 8 (1.7) | 10 (5.5) |  |  | | unknown | | 8 (0.8) | | 6 (0.6) | |  | | 5 (0.7) | | 2 (0.9) |  |
|  |  |  |  |  |  |  |  | |  | |  | |  | |  | |  | |  |  |
| ***Average lifetime of ethanol intake per day (g/day)*** | | | | | | | | | | | | | | | | | | | | |
| none | 251 (33.6) | 207 (28.3) | 0.05 | 150 (31.1) | 71 (38.8) | 0.40 |  | | none | | 44 (4.2) | | 58 (5.4) | | 0.0003 | | 29 (4.0) | | 11 (4.8) | 0.56 |
| <3.1 | 150 (20.1) | 132 (18.0) |  | 99 (20.5) | 36 (19.7) |  |  | | 0< to <8.4 | | 183 (17.4) | | 252 (23.4) | |  | | 124 (16.9) | | 36 (15.9) |  |
| ≥3.1 to <6.0 | 122 (16.4) | 134 (18.3) |  | 84 (17.4) | 25 (13.7) |  |  | | ≥8.4 to <17.6 | | 256 (24.3) | | 258 (23.9) | |  | | 188 (25.7) | | 47 (20.7) |  |
| ≥6.0 to <10.9 | 102 (13.7) | 128 (17.5) |  | 67 (13.9) | 25 (13.7) |  |  | | ≥17.6 to <30.9 | | 239 (22.7) | | 256 (23.7) | |  | | 161 (22.0) | | 54 (23.8) |  |
| ≥10.9 | 119 (16.0) | 131 (17.9) |  | 80 (16.6) | 26 (14.2) |  |  | | ≥30.9 | | 324 (30.8) | | 254 (23.6) | |  | | 226 (30.9) | | 77 (33.9) |  |
| unknown | 2 (0.3) | 0 (0.0) |  | 2 (0.4) | 0 (0.0) |  |  | | unknown | | 6 (0.6) | | 0 (0.0) | |  | | 4 (0.5) | | 2 (0.9) |  |
|  |  |  |  |  |  |  |  | |  | |  | |  | |  | |  | |  |  |
| ***Average physical activity in metabolic equivalents of task in last 12 months (hours/week)*** | | | | | | | | | | | | | | | | | | | | |
| <84.6 | 223 (29.9) | 181 (24.7) | 0.07 | 126 (26.1) | 62 (33.9) | 0.08 |  | | <53.6 | | 234 (22.2) | | 262 (24.3) | | 0.07 | | 154 (21.0) | | 58 (25.6) | 0.48 |
| ≥84.6 to <122.5 | 164 (22.0) | 178 (24.3) |  | 115 (23.9) | 33 (18.0) |  |  | | ≥53.6 to <99.4 | | 224 (21.3) | | 268 (24.9) | |  | | 155 (21.2) | | 49 (21.6) |  |
| ≥122.5 to <183.0 | 162 (21.7) | 181 (24.7) |  | 119 (24.7) | 34 (18.6) |  |  | | ≥99.4 to <167.1 | | 266 (25.3) | | 267 (24.8) | |  | | 188 (25.7) | | 53 (23.3) |  |
| ≥183.0 | 164 (22.0) | 185 (25.3) |  | 109 (22.6) | 40 (21.9) |  |  | | ≥167.1 | | 306 (29.1) | | 270 (25.0) | |  | | 221 (30.2) | | 62 (27.3) |  |
| unknown | 33 (4.4) | 7 (1.0) |  | 13 (2.7) | 14 (7.7) |  |  | | unknown | | 22 (2.1) | | 11 (1.0) | |  | | 14 (1.9) | | 5 (2.2) |  |
|  |  |  |  |  |  |  |  | |  | |  | |  | |  | |  | |  |  |
| ***Average lifetime pack years of regular smoking*** | | | | | | | | | | | | | | | | | | | | |
| non-smoker | 517 (69.3) | 526 (71.9) | 0.41 | 332 (68.9) | 134 (73.2) | 0.57 |  | | non-smoker | | 344 (32.7) | | 417 (38.7) | | 0.004 | | 237 (32.4) | | 78 (34.4) | 0.53 |
| >0 to <10 | 93 (12.5) | 90 (12.3) |  | 69 (14.3) | 18 (9.8) |  |  | | >0 to <10 | | 193 (18.3) | | 221 (20.5) | |  | | 142 (19.4) | | 33 (14.5) |  |
| ≥10 to <20 | 55 (7.4) | 46 (6.3) |  | 33 (6.8) | 15 (8.2) |  |  | | 10 to <20 | | 163 (15.5) | | 156 (14.5) | |  | | 111 (15.2) | | 40 (17.6) |  |
| ≥20 to <30 | 37 (5.0) | 39 (5.3) |  | 22 (4.6) | 8 (4.4) |  |  | | 20 to <30 | | 150 (14.3) | | 121 (11.2) | |  | | 102 (13.9) | | 32 (14.1) |  |
| ≥30 | 43 (5.8) | 28 (3.8) |  | 25 (5.2) | 8 (4.4) |  |  | | ≥30 | | 189 (18.0) | | 154 (14.3) | |  | | 130 (17.8) | | 42 (18.5) |  |
| unknown | 1 (0.1) | 3 (0.4) |  | 1 (0.2) | 0 (0.0) |  |  | | unknown | | 13 (1.2) | | 9 (0.8) | |  | | 10 (1.4) | | 2 (0.9) |  |
|  |  |  |  |  |  |  |  | |  | |  | |  | |  | |  | |  |  |
| ***History of colorectal cancer in a first degree relative*** | | | | | | | | | | | | | | | | | | | | |
| no | 608 (81.5) | 612 (83.6) | 0.19 | 392 (81.3) | 148 (80.9) | 0.26 |  | | no | | 880 (83.7) | | 942 (87.4) | | 0.01 | | 621 (84.8) | | 185 (81.5) | 0.89 |
| yes | 116 (15.5) | 96 (13.1) |  | 81 (16.8) | 23 (12.6) |  |  | | yes | | 143 (13.6) | | 106 (9.8) | |  | | 97 (13.3) | | 28 (12.3) |  |
| unknown | 22 (2.9) | 24 (3.3) |  | 9 (1.9) | 12 (6.5) |  |  | | unknown | | 29 (2.8) | | 30 (2.8) | |  | | 14 (1.9) | | 14 (6.1) |  |
|  |  |  |  |  |  |  |  | |  | |  | |  | |  | |  | |  |  |
| ***Diagnosis of diabetes ever (through a physician)*** | | | | | | | | | | | | | | | | | | | | |
| no | 598 (80.2) | 646 (88.3) | 0.0001 | 394 (81.7) | 141 (77.0) | 0.24 |  | | no | | 858 (81.6) | | 912 (84.6) | | 0.07 | | 608 (83.1) | | 177 (78.0) | 0.06 |
| yes | 138 (18.5) | 85 (11.6) |  | 82 (17.0) | 38 (20.8) |  |  | | yes | | 188 (17.9) | | 162 (15.0) | |  | | 121 (16.5) | | 50 (22.0) |  |
| unknown | 10 (1.4) | 1 (0.1) |  | 6 (1.2) | 4 (2.2) |  |  | | unknown | | 6 (0.6) | | 4 (0.4) | |  | | 3 (0.4) | | 0 (0.0) |  |
|  |  |  |  |  |  |  |  | |  | |  | |  | |  | |  | |  |  |
| ***Ever colorectal endoscopy*** | | | | | | | | | | | | | | | | | | | | |
| no | 599 (80.3) | 351 (48.0) | <0.0001 | 388 (80.5) | 147 (80.3) | 0.92 |  | | no | | 825 (78.4) | | 498 (46.2) | | <0.0001 | | 578 (79.0) | | 185 (81.5) | 0.41 |
| yes | 146 (19.6) | 381 (52.0) |  | 93 (19.3) | 36 (19.7) |  |  | | yes | | 227 (21.6) | | 580 (53.8) | |  | | 154 (21.0) | | 42 (18.5) |  |
| unknown | 1 (0.1) | 0 (0.0) |  | 1 (0.2) | 0 (0.0) |  |  | | unknown | | 0 (0.0) | | 0 (0.0) | |  | | 0 (0.0) | | 0 (0.0) |  |
|  |  |  |  |  |  |  |  | |  | |  | |  | |  | |  | |  |  |
| ***Ever regular use of non-steroidal anti-inflammatory drugs 2+ times/week ≥1 year*** | | | | | | | | | | | | | | | | | | | | |
| no | 573 (76.8) | 518 (70.8) | 0.01 | 414 (76.7) | 155 (76.7) | 0.95 |  | | no | | 812 (77.2) | | 733 (68.0) | | <0.01 | | 608 (77.2) | | 199 (78.3) | 0.64 |
| yes | 171 (22.9) | 210 (28.7) |  | 124 (23.0) | 47 (23.3) |  |  | | yes | | 234 (22.2) | | 342 (31.7) | |  | | 176 (22.3) | | 53 (20.9) |  |
| unknown | 2 (0.3) | 4 (0.5) |  | 2 (0.4) | 0 (0.0) |  |  | | unknown | | 6 (0.6) | | 3 (0.3) | |  | | 4 (0.5) | | 2 (0.8) |  |
|  |  |  |  |  |  |  |  | |  | |  | |  | |  | |  | |  |  |
| ***Consumption of red meat in categories*** | | | | | | | | | | | | | | | | | | | | |
| low | 98 (13.1) | 120 (16.4) | 0.06 | 68 (12.6) | 30 (14.9) | 0.47 |  | | low | | 48 (4.6) | | 72 (6.7) | | <0.01 | | 38 (4.8) | | 10 (3.9) | 0.77 |
| moderate | 617 (82.7) | 595 (81.3) |  | 451 (83.5) | 162 (80.2) |  |  | | moderate | | 905 (86.0) | | 946 (87.8) | |  | | 677 (85.9) | | 218 (85.8) |  |
| high | 29 (3.9) | 17 (2.3) |  | 19 (3.5) | 10 (5.0) |  |  | | high | | 99 (9.4) | | 60 (5.6) | |  | | 73 (9.3) | | 26 (10.2) |  |
| unknown | 2 (0.3) | 0 (0.0) |  | 2 (0.4) | 0 (0.0) |  |  | | unknown | | 0 (0.0) | | 0 (0.0) | |  | | 0 (0.0) | | 0 (0.0) |  |
|  |  |  |  |  |  |  |  | |  | |  | |  | |  | |  | |  |  |
| ***Ever use of menopausal hormone therapy^b^*** | | | | | | | | | | | | | | | | | | | | |
| no | 460 (67.2) | 340 (49.7) | <0.0001 | 279 (63.4) | 135 (78.5) | 0.0003 |  | | no | | n/a | | n/a | | n/a | | n/a | | n/a | n/a |
| yes | 214 (31.2) | 335 (49.0) |  | 153 (34.8) | 34 (19.8) |  |  | | yes | | n/a | | n/a | |  | | n/a | | n/a |  |
| unknown | 11 (1.6) | 9 (1.3) |  | 8 (1.8) | 3 (1.7) |  |  | | unknown | | n/a | | n/a | |  | | n/a | | n/a |  |
|  |  |  |  |  |  |  |  | |  | |  | |  | |  | |  | |  |  |
| ***UICC cancer stage*** | | | | | | | | | | | | | | | | | | | | |
| stage I | 164 (22.0) | n/a | n/a | 133 (27.6) | 12 (6.6) | <0.0001 |  | | stage I | | 271 (25.8) | | n/a | | n/a | | 217 (29.6) | | 22 (9.7) | <0.0001 |
| stage II | 235 (31.5) | n/a |  | 167 (34.6) | 44 (24.0) |  |  | | stage II | | 306 (29.1) | | n/a | |  | | 244 (33.3) | | 39 (17.2) |  |
| stage III | 247 (33.1) | n/a |  | 161 (33.4) | 54 (29.5) |  |  | | stage III | | 320 (30.4) | | n/a | |  | | 225 (30.7) | | 67 (29.5) |  |
| stage IV | 99 (13.3) | n/a |  | 21 (4.4) | 72 (39.3) |  |  | | stage IV | | 152 (14.4) | | n/a | |  | | 44 (6.0) | | 98 (43.2) |  |
| unknown | 1 (0.1) | n/a |  | 0 (0.0) | 1 (0.5) |  |  | | unknown | | 3 (0.3) | | n/a | |  | | 2 (0.3) | | 1 (0.4) |  |
|  |  |  |  |  |  |  |  | |  | |  | |  | |  | |  | |  |  |
| ***Tumour localization*** | | | | | | | | | | | | | | | | | | | | |
| colon | 500 (67.0) | n/a | n/a | 322 (66.8) | 126 (68.9) | 0.62 |  | | colon | | 597 (56.7) | | n/a | | n/a | | 406 (55.5) | | 132 (58.1) | 0.48 |
| rectum | 246 (33.0) | n/a |  | 160 (33.2) | 57 (31.1) |  |  | | rectum | | 455 (43.3) | | n/a | |  | | 326 (44.5) | | 95 (41.9) |  |
|  |  |  |  |  |  |  |  | |  | |  | |  | |  | |  | |  |  |
| **ESR2 *status*** | | | | | | | | | | | | | | | | | | | | |
| positive | 224 (30.0) | n/a | n/a | 159 (33.0) | 45 (24.6) | 0.002 |  | | positive | | 304 (28.9) | | n/a | | n/a | | 216 (29.5) | | 63 (27.8) | 0.35 |
| negative | 225 (30.2) | n/a |  | 124 (25.7) | 71 (38.8) |  |  | | negative | | 278 (26.4) | | n/a | |  | | 190 (26.0) | | 67 (29.5) |  |
| unknown | 297 (38.8) | n/a |  | 199 (41.3) | 67 (36.6) |  |  | | unknown | | 470 (44.7) | | n/a | |  | | 326 (44.5) | | 97 (42.7) |  |
|  |  |  |  |  |  |  |  | |  | |  | |  | |  | |  | |  |  |
| ***AR* CAG(n)** | | | | | | | | | | | | | | | | | | | | |
| <22/<22 repeats | 168 (22.5) | 172 (23.5) | 0.56 | 107 (22.2) | 41 (22.3) | 0.69 |  | | <22/<22 repeats | | 477 (45.3) | | 438 (40.6) | | 0.07 | | 322 (44.0) | | 111 (48.9) | 0.31 |
| <22/≥22 repeats | 324 (43.4) | 324 (44.3) |  | 205 (42.5) | 82 (44.8) |  |  | | <22/≥22 repeats | | n/a | | n/a | |  | | n/a | | n/a |  |
| ≥22/≥22 repeats | 176 (23.6) | 155 (21.2) |  | 121 (25.1) | 40 (21.9) |  |  | | ≥22/≥22 repeats | | 443 (42.1) | | 483 (44.8) | |  | | 315 (43.0) | | 92 (40.5) |  |
| unknown | 78 (10.5) | 81 (11.1) |  | 49 (10.2) | 20 (10.9) |  |  | | Unknown | | 132 (12.6) | | 157 (14.6) | |  | | 95 (13.0) | | 24 (10.6) |  |
|  |  |  |  |  |  |  |  | |  | |  | |  | |  | |  | |  |  |
| ***ESR2* CA(n)** | | | | | | | | | | | | | | | | | | | | |
| <24/<24 repeats | 119 (16.0) | 125 (17.1) | 0.73 | 73 (15.1) | 31 (16.9) | 0.83 |  | | <24/<24 repeats | | 204 (19.4) | | 222 (20.6) | | 0.08 | | 134 (18.3) | | 48 (21.1) | 0.52 |
| <24/≥24 repeats | 335 (44.9) | 334 (45.6) |  | 219 (45.4) | 80 (43.7) |  |  | | <24/≥24 repeats | | 420 (39.9) | | 480 (44.5) | |  | | 294 (40.2) | | 86 (37.9) |  |
| ≥24/≥24 repeats | 208 (27.8) | 193 (26.4) |  | 138 (28.6) | 51 (27.9) |  |  | | ≥24/≥24 repeats | | 295 (28.1) | | 265 (24.6) | |  | | 213 (29.1) | | 60 (26.4) |  |
| unknown | 84 (11.3) | 80 (10.9) |  | 52 (10.8) | 21 (11.5) |  |  | | Unknown | | 133 (12.6) | | 111 (10.3) | |  | | 91 (12.4) | | 33 (14.5) |  |
|  |  |  |  |  |  |  |  |  | |  | |  | |  | |  | |  | |  |
| n/a: characteristic only available for cases or women  ^a^Among patients eligible for survival analysis  ^b^Among the postmenopausal female study population | | | | | | | | | | | | | | | | | | | | |

**Table S2:** Number of CAG repeats in *AR* and CA repeats in *ESR2* and associated risk for ESR2 positive and ESR2 negative colorectal cancer in the female and male study population.

|  | **Overall** | | | **Women** | | | **Men** | | |  |
| --- | --- | --- | --- | --- | --- | --- | --- | --- | --- | --- |
| **Genotype** | **Cases N** | **Controls N** | **OR (95% CI)^b^** | **Cases N** | **Controls N** | **OR (95% CI)^c^** | **Cases N** | **Controls N** | **OR (95% CI)^c^** | ***p* heterogeneity** |
|  |  |  |  |  |  |  |  |  |  |  |
| ***AR* CAG(n)** |  |  |  |  |  |  |  |  |  |  |
| ***ESR2 positive*** |  |  |  |  |  |  |  |  |  |  |
| <20 average repeats | 78 | 254 | 1.00 (Ref.) | 25 | 94 | 1.00 (Ref.) | 53 | 160 | 1.00 (Ref.) |  |
| ≥20 to <22 average repeats | 158 | 506 | 1.00 (0.74 - 1.37) | 72 | 228 | 1.15 (0.69 - 1.94) | 86 | 278 | 0.91 (0.61 - 1.36) |  |
| ≥22 to <23.5 average repeats | 114 | 400 | 0.91 (0.66 - 1.27) | 53 | 189 | 1.03 (0.60 - 1.77) | 61 | 211 | 0.87 (0.57 - 1.33) |  |
| ≥23.5 average repeats | 127 | 412 | 0.98 (0.71 - 1.35) | 53 | 140 | 1.32 (0.76 - 2.28) | 74 | 272 | 0.80 (0.53 - 1.20) | 0.62^d^ |
| average repeats^a^ | 477 | 1572 | 1.00 (0.96 - 1.04) | 203 | 651 | 1.02 (0.94 - 1.09) | 274 | 921 | 0.99 (0.94 - 1.03) | 0.43^e^ |
|  |  |  | *p* trend = 0.85 |  |  | *p* trend = 0.55 |  |  | *p* trend = 0.55 |  |
|  |  |  |  |  |  |  |  |  |  |  |
| <22/<22 repeats | 193 | 610 | 1.00 (Ref.) | 54 | 172 | 1.00 (Ref.) | 139 | 438 | 1.00 (Ref.) |  |
| <22/≥22 repeats | 96 | 324 | 0.87 (0.62 - 1.21) | 96 | 324 | 0.93 (0.63 - 1.37) | n/a | n/a | (no heterozygous) |  |
| ≥22/≥22 repeats | 188 | 638 | 0.92 (0.73 - 1.16) | 53 | 155 | 1.06 (0.68 - 1.65) | 135 | 483 | 0.88 (0.67 - 1.16) | 0.49^e^ |
|  |  |  | *p* trend = 0.49 |  |  | *p* trend = 0.82 |  |  | *p* trend = 0.36 |  |
|  |  |  |  |  |  |  |  |  |  |  |
| ***ESR2 negative*** |  |  |  |  |  |  |  |  |  |  |
| <20 average repeats | 73 | 254 | 1.00 (Ref.) | 28 | 94 | 1.00 (Ref.) | 45 | 160 | 1.00 (Ref.) |  |
| ≥20 to <22 average repeats | 161 | 506 | 1.11 (0.81 - 1.53) | 73 | 228 | 1.08 (0.65 - 1.78) | 88 | 278 | 1.14 (0.76 - 1.73) |  |
| ≥22 to <23.5 average repeats | 88 | 400 | 0.76 (0.53 - 1.08) | 46 | 189 | 0.81 (0.47 - 1.38) | 42 | 211 | 0.72 (0.45 - 1.16) |  |
| ≥23.5 average repeats | 129 | 412 | 1.11 (0.80 - 1.54) | 54 | 140 | 1.31 (0.77 - 2.23) | 75 | 272 | 0.99 (0.65 - 1.50) | 0.14^d^ |
| average repeats^a^ | 451 | 1572 | 0.99 (0.95 - 1.03) | 201 | 651 | 1.02 (0.95 - 1.10) | 250 | 921 | 0.97 (0.93 - 1.02) | 0.23^e^ |
|  |  |  | *p* trend = 0.53 |  |  | *p* trend = 0.66 |  |  | *p* trend = 0.24 |  |
|  |  |  | *p* het**^d^** = 0.54 |  |  | *p* het**^d^** = 0.87 |  |  | *p* het**^d^** = 0.53 |  |
|  |  |  |  |  |  |  |  |  |  |  |
| <22/<22 repeats | 185 | 610 | 1.00 (Ref.) | 52 | 172 | 1.00 (Ref.) | 133 | 438 | 1.00 (Ref.) |  |
| <22/≥22 repeats | 90 | 324 | 0.79 (0.56 - 1.10) | 90 | 324 | 0.93 (0.63 - 1.38) | n/a | n/a | (no heterozygous) |  |
| ≥22/≥22 repeats | 176 | 638 | 0.92 (0.72 - 1.16) | 59 | 155 | 1.29 (0.83 - 1.99) | 117 | 483 | 0.80 (0.60 - 1.06) | 0.08^e^ |
|  |  |  | *p* trend = 0.46 |  |  | *p* trend = 0.26 |  |  | *p* trend = 0.12 |  |
|  |  |  | *p* het**^d^** = 0.87 |  |  | *p* het**^d^** = 0.59 |  |  | *p* het**^d^** = 0.57 |  |
|  |  |  |  |  |  |  |  |  |  |  |
| ***ESR2* CA(n)** |  |  |  |  |  |  |  |  |  |  |
| ***ESR positive*** |  |  |  |  |  |  |  |  |  |  |
| <22 average repeats | 102 | 409 | 1.00 (Ref.) | 41 | 153 | 1.00 (Ref.) | 61 | 256 | 1.00 (Ref.) |  |
| ≥22 to <23.5average repeats | 107 | 381 | 1.15 (0.85 - 1.56) | 44 | 154 | 1.07 (0.66 - 1.75) | 63 | 227 | 1.18 (0.79 - 1.75) |  |
| ≥23.5 to <24 average repeats | 77 | 227 | 1.39 (0.99 - 1.95) | 32 | 90 | 1.36 (0.79 - 2.33) | 45 | 137 | 1.39 (0.90 - 2.16) |  |
| ≥24 average repeats | 179 | 602 | 1.19 (0.91 - 1.57) | 80 | 255 | 1.16 (0.75 - 1.79) | 99 | 347 | 1.19 (0.83 - 1.71) | 0.70^e^ |
| average repeats^a^ | 465 | 1619 | 1.05 (0.99 - 1.12) | 197 | 652 | 1.02 (0.93 - 1.13) | 268 | 967 | 1.06 (0.98 - 1.15) | 0.59^f^ |
|  |  |  | *p* trend = 0.12 |  |  | *p* trend = 0.64 |  |  | *p* trend = 0.13 |  |
|  |  |  |  |  |  |  |  |  |  |  |
| <24/<24 repeats | 95 | 347 | 1.00 (Ref.) | 33 | 125 | 1.00 (Ref.) | 62 | 222 | 1.00 (Ref.) |  |
| <24/≥24 repeats | 224 | 814 | 1.01 (0.77 - 1.32) | 102 | 334 | 1.16 (0.74 - 1.82) | 122 | 480 | 0.91 (0.64 - 1.29) |  |
| ≥24/≥24 repeats | 146 | 458 | 1.15 (0.86 - 1.55) | 62 | 193 | 1.20 (0.74 - 1.96) | 84 | 265 | 1.12 (0.77 - 1.64) | 0.82^f^ |
|  |  |  | *p* trend = 0.31 |  |  | *p* trend = 0.49 |  |  | *p* trend = 0.49 |  |
|  |  |  |  |  |  |  |  |  |  |  |
| ***ESR2 negative*** |  |  |  |  |  |  |  |  |  |  |
| <22 average repeats | 102 | 409 | 1.00 (Ref.) | 53 | 153 | 1.00 (Ref.) | 49 | 256 | 1.00 (Ref.) |  |
| ≥22 to <23.5average repeats | 103 | 381 | 1.07 (0.79 - 1.46) | 38 | 154 | 0.70 (0.43 - 1.13) | 65 | 227 | 1.46 (0.97 - 2.21) |  |
| ≥23.5 to <24 average repeats | 63 | 227 | 1.11 (0.78 - 1.58) | 24 | 90 | 0.75 (0.43 - 1.31) | 39 | 137 | 1.49 (0.93 - 2.39) |  |
| ≥24 average repeats | 173 | 602 | 1.15 (0.87 - 1.51) | 83 | 255 | 0.92 (0.61 - 1.37) | 90 | 347 | 1.37 (0.93 - 2.01) | 0.35^e^ |
| average repeats^a^ | 441 | 1619 | 1.04 (0.98 - 1.11) | 198 | 652 | 0.98 (0.89 - 1.07) | 243 | 967 | 1.08 (1.00 - 1.18) | 0.15^f^ |
|  |  |  | *p* trend = 0.22 |  |  | *p* trend = 0.62 |  |  | *p* trend = 0.06 |  |
|  |  |  | *p* het**^d^** = 0.79 |  |  | *p* het**^d^** = 0.53 |  |  | *p* het**^d^** = 0.81 |  |
|  |  |  |  |  |  |  |  |  |  |  |
| <24/<24 repeats | 94 | 347 | 1.00 (Ref.) | 42 | 125 | 1.00 (Ref.) | 52 | 222 | 1.00 (Ref.) |  |
| <24/≥24 repeats | 205 | 814 | 0.92 (0.70 - 1.22) | 89 | 334 | 0.77 (0.51 - 1.18) | 116 | 480 | 1.04 (0.72 - 1.50) |  |
| ≥24/≥24 repeats | 142 | 458 | 1.14 (0.85 - 1.54) | 67 | 193 | 1.00 (0.64 - 1.57) | 75 | 265 | 1.23 (0.83 - 1.83) | 0.71^f^ |
|  |  |  | *p* trend = 0.29 |  |  | *p* trend = 0.80 |  |  | *p* trend = 0.29 |  |
|  |  |  | *p* het**^d^** = 0.99 |  |  | *p* het**^d^** = 0.77 |  |  | *p* het**^d^** = 0.85 |  |
|  |  |  |  |  |  |  |  |  |  |  |
| ^a^As continuous variable, ^b^Models adjusted for sex, county of residence and age, ^c^Models adjusted for county of residence and age, ^d^*P*-value from case-only analysis for heterogeneity between risk estimates for ESR2 positive and ESR2 negative disease, ^e^*P*-value for heterogeneity by gender with genotype in categories (3df), ^f^*P*-value for heterogeneity by gender with genotype as continuous variable (1df), OR: odds ratio, CI: confidence interval | | | | | | | | | | |

**Table S3:** Associations between number of *AR* CAG repeats and *ESR2* CA repeats and overall as well as disease specific survival according to tumoral ESR2 expression.

|  | **ESR2 positive** | | **ESR2 negative** | | ***p* heterogeneity** | |
| --- | --- | --- | --- | --- | --- | --- |
| **Genotype** | **OS HR (95% CI)^b^** | **DSS HR (95% CI)^b^** | **OS HR (95% CI)^b^** | **DSS HR (95% CI)^b^** | **OS** | **DSS** |
|  |  |  |  |  |  |  |
| ***AR* CAG(n)** |  |  |  |  |  |  |
| <20 average repeats | 1.00 (Ref.) | 1.00 (Ref.) | 1.00 (Ref.) | 1.00 (Ref.) |  |  |
| ≥20 to <22 average repeats | 1.92 (0.91 - 4.02) | 2.00 (0.82 - 4.88) | 1.51 (0.86 - 2.67) | 1.52 (0.81 -2.87) |  |  |
| ≥22 to <23.5 average repeats | 2.37 (1.10 - 5.11) | 3.57 (1.44 - 8.83) | 1.05 (0.52 - 2.13) | 1.00 (0.44 - 2.28) |  |  |
| ≥23.5 average repeats | 1.62 (0.74 - 3.53) | 1.46 (0.55 - 3.85) | 1.60 (0.88 - 2.90) | 1.78 (0.91 - 3.49) | 0.24^c^ | 0.03^c^ |
| average repeats^a^ | 1.05 (0.97 - 1.14) | 1.06 (0.95 - 1.17) | 1.05 (0.97 - 1.13) | 1.06 (0.97 - 1.16) | 0.89^d^ | 0.83^d^ |
|  | *p* trend = 0.23 | *p* trend = 0.30 | *p* trend = 0.22 | *p* trend = 0.18 |  |  |
|  |  |  |  |  |  |  |
| <22/<22 repeats | 1.00 (Ref.) | 1.00 (Ref.) | 1.00 (Ref.) | 1.00 (Ref.) |  |  |
| <22/≥22 repeats | 1.05 (0.58 - 1.90) | 1.05 (0.51 - 2.14) | 1.28 (0.76 - 2.15) | 1.35 (0.73 - 2.51) |  |  |
| ≥22/≥22 repeats | 1.34 (0.80 - 2.22) | 1.88 (1.01 - 3.50) | 1.23 (0.78 - 1.96) | 1.44 (0.83 - 2.48) | 0.82^d^ | 0.53^d^ |
|  | *p* trend = 0.27 | *p* trend = 0.06 | *p* trend = 0.39 | *p* trend = 0.20 |  |  |
|  |  |  |  |  |  |  |
| ***ESR2* CA(n)** |  |  |  |  |  |  |
| <22 average repeats | 1.00 (Ref.) | 1.00 (Ref.) | 1.00 (Ref.) | 1.00 (Ref.) |  |  |
| ≥22 to <23.5average repeats | 0.91 (0.47 - 1.75) | 1.13 (0.50 - 2.53) | 1.53 (0.81 - 2.89) | 1.77 (0.84 - 3.72) |  |  |
| ≥23.5 to <24 average repeats | 0.83 (0.43 - 1.61) | 1.12 (0.51 - 2.46) | 1.68 (0.85 - 3.34) | 2.16 (0.96 - 4.84) |  |  |
| ≥24 average repeats | 0.88 (0.49 - 1.55) | 1.06 (0.52 - 2.18) | 0.87 (0.49 - 1.55) | 0.83 (0.42 - 1.65) | 0.36^c^ | 0.26^c^ |
| average repeats^a^ | 0.98 (0.86 - 1.12) | 0.98 (0.84 - 1.15) | 0.98 (0-87 - 1.11) | 0.98 (0.85 - 1.12) | 0.87^d^ | 0.83^d^ |
|  | *p* trend = 0.76 | *p* trend = 0.85 | *p* trend = 0.77 | *p* trend = 0.72 |  |  |
|  |  |  |  |  |  |  |
| <24/<24 repeats | 1.00 (Ref.) | 1.00 (Ref.) | 1.00 (Ref.) | 1.00 (Ref.) |  |  |
| <24/≥24 repeats | 0.79 (0.45 - 1.38) | 0.74 (0.38 - 1.45) | 0.94 (0.56 - 1.57) | 1.43 (0.77 - 2.66) |  |  |
| ≥24/≥24 repeats | 0.93 (0.50 - 1.74) | 1.01 (0.49 - 2.08) | 0.63 (0.36 - 1.11) | 0.67 (0.34 - 1.35) | 0.51^d^ | 0.46^d^ |
|  | *p* trend = 0.86 | *p* trend = 0.89 | *p* trend = 0.10 | *p* trend = 0.23 |  |  |
|  |  |  |  |  |  |  |
| ^a^As continuous variable, ^b^Stratified for grade of differentiation (well/moderate, poor/undifferentiated) and adjusted for diagnosis of other cancer after colorectal cancer diagnosis (yes/no), colorectal cancer detected by screening (yes/no), treatment with adjuvant chemotherapy (yes/no), tumor extent (T1, T2, T3, T4), nodal status (N0, N1, N2), distant metastasis (M0, M1), BMI (kg/m^2^, continuous), age at diagnosis and year of diagnosis, ^c^*P*-value for heterogeneity by ESR2 status with genotype in categories (3df), ^d^*P*-value for heterogeneity by ESR2 status with genotype as continuous variable (1df), OS: overall survival, DSS: disease-specific survival, HR: hazard ratio, CI: confidence interval | | | | | | |
